# Society Transactions

**Published:** 1889-08

**Authors:** 


### SOCIETY TRANSACTIONS:

I. **Transactions of the Medical Society of the State of New York for the Year 1889.** Published by the Society. Standard 8vo, pp. 507. William J. Dornan, printer. 1889.

II. **Transactions of the New York State Medical Association for the Year 1888.** Volume V. Edited for the Association by ALFRED LUDLOW CARROLL, M. D. Small 8vo, pp. 610. Concord, N. H.: Republican Press Association. New York: J. H. Vail & Co. 1889.

III. **Transactions of the American Gynecological Society.** Volume XIII. For the Year 1888. Small 8vo; pp. 502. Philadelphia: William J. Dornan. 1888.

I. Never since the Society assumed the publication of its Transactions, in 1875, has its annual volume appeared more promptly or in better form. The paper, press-work, and binding are very much improved, and, taken altogether, it is an agreeable book to handle and to look upon. Its contents, too, exhibit much careful work of scientific value. Some of the papers are of more than ordinary excellence, and deserve thoughtful study, while all are of a nature to reflect credit upon their authors and upon the Society that puts forth the volume as well. We have so recently (see March number) discoursed upon the work of the Society at this meeting, that it is unnecessary to go into further detail here.

II. The Transactions of the New York State Medical Association is an ornamental book, and contains many papers of professional interest and scientific excellence. Some of the discussions are of especial value, and deserve to be preserved in this neat and durable form. We observe that many physicians take active interest in both these State organizations,—a gratifying sign of harmony and professional good-fellowship.

III. The Transactions of the American Gynecological Society presents its usual fine appearance, and the volume is replete with interesting material. The Transactions of the special societies for 1888 possess an added interest from the fact that it was the first year of the Congress of American Physicians and Surgeons, and so a large attendance was attracted to the meetings, and hence the work done was, generally speaking, of more than usual quality. This Society is no exception to the rule, though it will not become a part of the Congress until 1891, when the latter convenes again. We think it would be well if these volumes of the Transactions of the special societies could be made to find their way into the hands of the profession more generally. The thirteen volumes of this Society's work that we happen to possess are among the highest prized of any equal number of books upon our shelves, and we would regard it a sad parting to separate from them.

